# FOLLOW-UP OF A 6-MONTH LOW-TO-MODERATE INTENSITY VIRTUAL-HOME EXERCISE PROGRAM TO PREVENT FALLS

**DOI:** 10.1093/geroni/igz038.2273

**Published:** 2019-11-08

**Authors:** Machiko R Tomita, Nadine M Fisher

**Affiliations:** University at Buffalo, Buffalo, New York, United States

## Abstract

The aim of this study is to determine 3-month post effects of and adherence to a 6-month virtual-group exercise at home (V-GEAH) program, which offered low-to-moderate intensity exercise to community-dwelling older adults with past falls. The V-GEAH program converted solitary exercise to group exercise connecting participants via web-conference technology. A treatment group (n=25, 60 – 90 years old) exercised three times a week for 30-45 minutes each session. The program achieved 84.4% – 93.3% adherence, reducing fall risks. This study measured falls, balance confidence, lower extremity muscle strength and endurance, gait speed, stride length, and activities of daily living, and compared with baseline and posttest (at 6 months) data using Repeated measures ANOVA with contrasts. During the follow-up period, 40% of the treatment group exercised 2+ times/week (Adherer, or A), and 60% did 1 time/week or less (Non-adherer or NA). Half of NA joined a community exercise group and the rest did not do exercise due to pain in various body parts. None of A fell while 26.7% of NA and 20% of C did. At follow-up, the control group (C, n=25) showed no change or significant decline from posttest for all measures. A maintained gains made in the intervention period in all measures, but NA significantly lost strength in hamstrings, hip abduction, and quadriceps and hamstring endurance. These results indicate that low-to-moderate intensity exercise and technology use for providing visual instruction, regular monitoring and evaluation, and environments to increase participants’ accountability are elements for successful home-based exercise programs.

